# Flt3 Does Not Play a Critical Role in Murine Myeloid Leukemias Induced by *MLL* Fusion Genes

**DOI:** 10.1371/journal.pone.0072261

**Published:** 2013-08-16

**Authors:** Stéphanie Albouhair, Ester Morgado, Catherine Lavau

**Affiliations:** Centre National de la Recherche Scientifique, UMR7151, Paris, France; Westmead Millennium Institute, University of Sydney, Australia

## Abstract

Leukemias harboring *MLL* translocations are frequent in children and adults, and respond poorly to therapies. The receptor tyrosine kinase FLT3 is highly expressed in these leukemias. *In vitro* studies have shown that pediatric *MLL*-rearranged ALL cells are sensitive to FLT3 inhibitors and clinical trials are ongoing to measure their therapeutic efficacy. We sought to determine the contribution of Flt3 in the pathogenesis of *MLL*-rearranged leukemias using a myeloid leukemia mouse model. Bone marrow from *Flt3* null mice transduced with *MLL-ENL* or *MLL-CBP* was transplanted into host mice and *Flt3* ^−/−^ leukemias were compared to their *Flt3* wild type counterparts. *Flt3* deficiency did not delay disease onset and had minimal impact on leukemia characteristics. To determine the anti-leukemic effect of FLT3 inhibition we studied the sensitivity of *MLL-ENL* leukemia cells to the FLT3 inhibitor PKC412 *ex vivo*. As previously reported for human *MLL*-rearranged leukemias, murine *MLL-ENL* leukemia cells with higher Flt3 levels were more sensitive to the cytotoxicity of PKC412. Interestingly, *Flt3* deficient leukemia samples also displayed some sensitivity to PKC412. Our findings demonstrate that myeloid leukemias induced by *MLL*-rearranged genes are not dependent upon Flt3 signaling. They also highlight the discrepancy between the sensitivity of cells to Flt3 inhibition *in vitro* and the lack of contribution of Flt3 to the pathogenesis of *MLL*-rearranged leukemias *in vivo*.

## Introduction

Chromosomal translocations involving the *Mixed Lineage Leukemia* (*MLL*) gene on chromosome 11q23 are present in approximately 10% of acute leukemias (myeloid- AML or lymphoblastic -ALL). These *MLL*-rearranged leukemias generally have a poor prognosis and a high rate of relapse following therapy. *MLL* translocations are particularly prevalent in infant leukemias and in secondary chemotherapy-related leukemias. In these two situations the disease latency is strikingly brief; the average age for diagnosis of infant leukemias is approximately 6 months and secondary leukemias can be detected 26 months after beginning of chemotherapy. This rapid disease onset suggests that *MLL* fusion genes are powerful oncogenes that, given the role of MLL in chromatin structure, could have pleiotropic effects and alter both signaling and differentiation pathways by having a global impact on gene expression [1]. This hypothesis finds support from gene expression profiling analyses that identified a gene signature distinguishing *MLL*-rearranged acute leukemias from other ALLs or AMLs [2]–[5]. This *MLL*-rearranged leukemia signature includes the *HOXA* cluster genes and the HOX cofactor *MEIS1* gene which are direct transcriptional targets of MLL [6]. Another gene specifically upregulated in *MLL*-rearranged leukemias that has been proposed to play a critical role in their pathogenesis is the gene encoding Fms-like tyrosine kinase 3 (FLT3) [3], [7]–[9]. Excessive expression of FLT3 in *MLL*-rearranged leukemias results from direct transcriptional activation by HOXA9 and MEIS1 [10] and post-transcriptional regulation by miR-150, a microRNA whose level is repressed by *MLL* fusion genes [11]. FLT3 is normally expressed in immature hematopoietic progenitors and, upon activation by its ligand, triggers signal transduction pathways that stimulate cell survival and proliferation. The upregulation of FLT3 and autocrine or paracrine activation by its ligand can lead to excessive FLT3 signaling in leukemia [12].

Mutations that constitutively activate FLT3, either internal tandem duplication in the juxtamembrane region or point mutations in the kinase domain, are detected in approximately one third of AMLs and confer a poor prognosis. Several small-molecule inhibitors have been developed to target FLT3 signaling and are being evaluated in clinical trials to treat AML patients [13]. The selective upregulation of FLT3 in *MLL*-rearranged ALLs raised the possibility that FLT3 inhibitors could also be beneficial in the treatment of these aggressive leukemias [2]. Two studies have shown that cultured leukemia cells from pediatric *MLL*-rearranged ALLs are more sensitive to the cytotoxicity of FLT3 inhibitors, PKC412 or CEP-701, than ALL cells with germline *MLL* [14], [15] expressing lower levels of FLT3. These results confirmed previous work showing the sensitivity of *MLL*-rearranged cell lines to PKC412 [7]. Clinical trials of FLT3 inhibitors, PKC412 and lestaurtinib, to treat pediatric *MLL*-rearranged ALL are ongoing [16].

Murine models to determine the role of Flt3 in the pathogenesis of *MLL*-rearranged leukemias have shown contradictory results. Using Flt3 knock out mice, we previously found that leukemias induced by the retroviral transduction of *Hoxa9* and *Meis1*, which recapitulate many features of *MLL*-rearranged myeloid leukemias, are Flt3 independent [17]. In contrast, a recent study showed that shRNA-mediated knock-down of Flt3 increases the latency of *MLL-AF9* induced leukemias in transplanted mice [11], supporting the notion that Flt3 is a worthy drug target. This could, however, not be validated in an earlier study showing that therapeutic inhibition of Flt3 by PKC412 does not reduce tumor burden of *MLL-AF9* leukemia in mice [18]. Here, we examine the role of Flt3 in the pathogenesis of *MLL*-rearranged myeloid leukemias by transducing two *MLL* fusion genes into *Flt3*-deficient bone marrow cells. We show that *Flt3*-null cells can be transformed by *MLL-ENL* or *MLL-CBP in vitro*. Moreover, following transplantation into recipient mice, *Flt3*-null cells induce similar leukemias to their *Flt3* wild type counterparts, providing evidence that Flt3 is dispensable to the genesis of leukemias induced by *MLL-ENL* or *MLL-CBP*. To explore whether Flt3 signaling in leukemia cells is important *ex vivo*, we studied the sensitivity of cultured primary *MLL-ENL* leukemia blasts to the FLT3 inhibitor PKC412. As was reported for human *MLL*-rearranged blasts, leukemia cells expressing higher levels of Flt3 are more sensitive to the cytotoxicity of PKC412. We also find that PKC412’s cytotoxicity involves off target activity. Altogether, our findings suggest that Flt3 requirements may differ for survival of *MLL*-rearranged leukemia cells *in vitro* and leukemogenesis *in vivo*.

## Materials and Methods

### Ethics Statement

Mouse care and experimental procedure were performed in accordance with the animal care and use committee of the Institut Universitaire d'Hématologie and approved protocols from the Comité Régional d'Ethique en Expérimentation Animale n°4.

### Mice and DNA Constructs

Homozygous *Flt3* ^−/−^ mice were kindly provided by Ihor Lemischka (Princeton University) and control wild-type (*Flt3* ^+/+^) 129/Sv mice were purchased from Charles River (Arbresle, France). MSCV*MLL-ENL*, MSCV*MLL-CBP,* MIE, MIE*MLL-ENL*, and MIE*MLL-CBP* constructs have been described previously [19], [20].

### Retroviral Transduction of Bone Marrow Progenitors and Transformation Assay *in vitro*

Production of retroviral supernatants and transduction of bone marrow (BM) Lin^neg^ progenitors were performed as previously described [21].

### Transplantation of Mice and Characterization of Leukemias

Eight to twelve-week-old lethally irradiated (2 split doses of 500 rad or a single dose of 900 rad) 129/Sv mice were transplanted by tail vein injection of 60–80×10^3^ infected progenitors with a radioprotective dose of 10^5^ syngenic BM cells. Blood cell counts were performed with a Cell-Dyn 3700 counter (Abbott Diagnostic, Rungis, France). Mice with signs of disease were euthanized and their tissues (spleen, liver, muscle and kidneys) were harvested for histological analyses. BM cytospins were stained with Wright-Giemsa for morphological analyses. BM, peripheral blood and spleen cell suspensions were blocked with rat IgG (10 µg/ml) prior to staining with fluorochrome-conjugated monoclonal antibodies specific for Mac-1, Gr-1, F4/80 or c-Kit (purchased from BD Biosciences or eBioscience) and analyzed by flow cytometry. Flt3 expression was analyzed following preincubation with the blocking anti CD16/CD32 antibody (eBioscience). Dead cells were excluded by propidium iodide staining. Fluorescence was analyzed on a LSR II (BD bioscience, San Jose, CA), using CellQuest software (BD bioscience).

Transplantability of tumors was assessed by tail vein injection of 10^5^ nucleated BM cells from a leukemic mouse into 2–4 non-irradiated immunodeficient *Rag2* ^−/−^ recipient mice.

### Protein Expression Analyses

Whole cell lysates were prepared from 5×10^5^ BM cells for Western blot analyses. A 1∶5000 dilution of anti-FLT3 SC-340 (Santa Cruz Biotechnology, Santa Cruz, CA) was used as the primary antibody, followed by rabbit peroxydase-conjugated secondary antibody and visualization using the enhanced chemiluminescence (ECL) reagent (Amersham). Hybridization with the rabbit polyclonal antibody to actin 20–33 (Sigma-Aldrich) was used as loading control.

### Cytotoxicity Assays of Primary Cells

PKC412 (Novartis, Basel, Switzerland) was dissolved in DMSO to make initial stock solutions and then diluted in media to obtain desired final concentrations. DMSO frozen BM cells harvested from secondary or tertiary transplanted *MLL-ENL* leukemic mice were used to study the sensitivity of primary leukemia samples to PKC412. These BM samples consistently contained more than 95% percent of GFP-expressing leukemic blasts. Thawed cells were seeded at high density (8×10^5^ cells/ml) and cultured for 48 h in RPMI 1640 medium with 20% FCS and supplements (50 U/mL penicillin G, 50 mg/mL streptomycin, 2 mM L-glutamine and 0.05 mM 2-mercaptoethanol), without recombinant growth factors. Cells were then seeded in 96 well plates at a density of 6–8×10^5^ cells/mL with various concentrations of PKC412 in duplicates or triplicates wells. After 3 days, viable cell numbers were determined by scoring trypan blue negative cells with a hemacytometer. Cell viability was reported as percentage of untreated cells. Samples for which the number of viable cells in the untreated wells dropped by more than 70% over the 3-day culture were not analyzed. The cytotoxicity response of primary leukemic cells was also evaluated using a XTT assay according to the manufacturer’s instruction (Roche).

### Statistical Analyses

Mice survival curves were compared using the log-rank test. Features of leukemias from the different cohorts were compared using the unpaired t test. Comparisons between untreated and PKC412-treated blasts were done using the paired t test. All statistical tests were performed with Prism 4.0 (GraphPad).

## Results

### *Flt3* is Dispensable for the Transforming Properties of *MLL-ENL* or *MLL-CBP in vitro*

**Figure 1:**
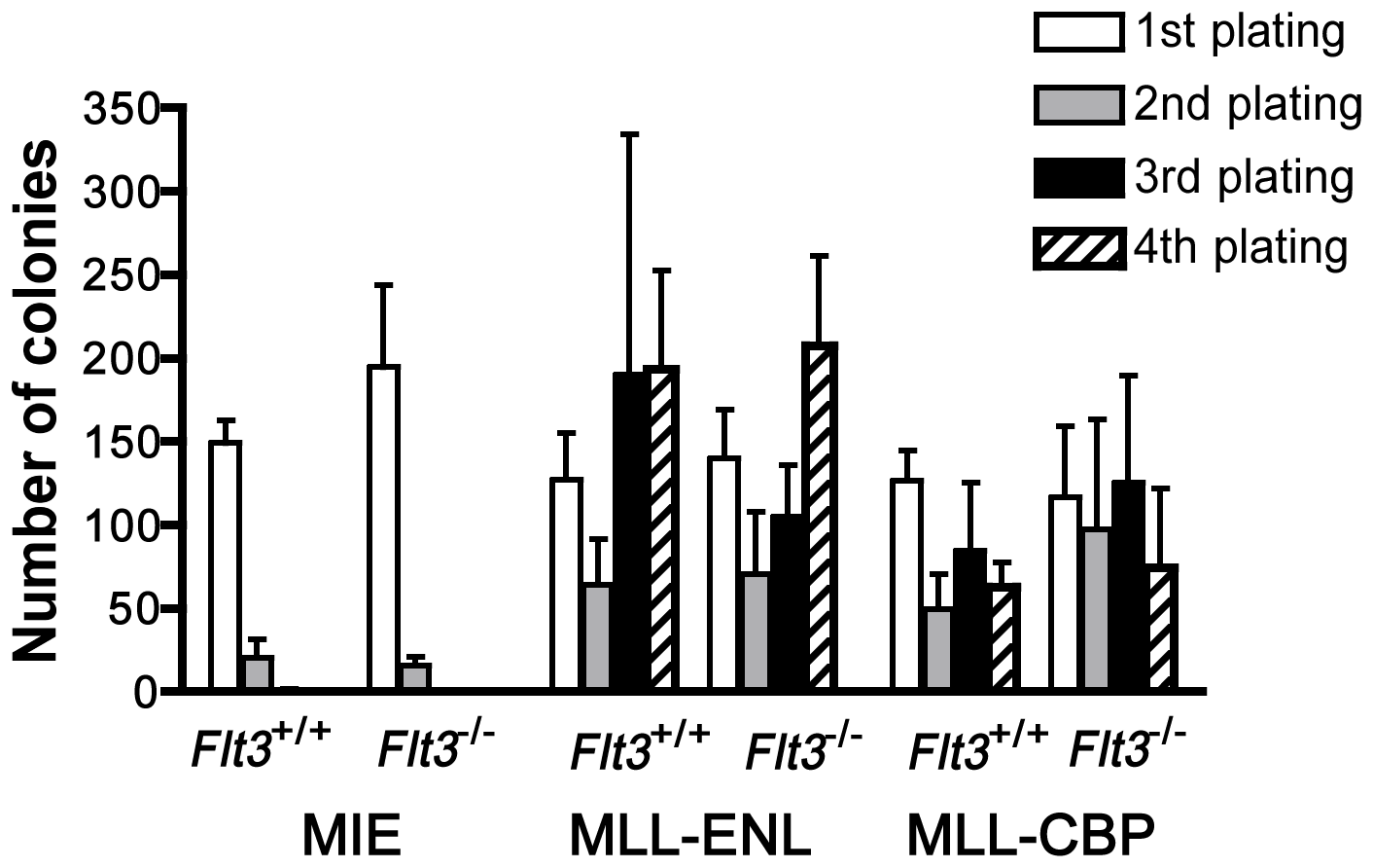
*MLL* fusion genes transform BM progenitors independently of *Flt3 in vitro*. Myeloid colony forming assays were performed with *Flt3* ^+/+^ and *Flt3* ^−/−^ progenitors infected by empty MIE vector, *MLL-ENL* or *MLL-CBP*-encoding vectors. Numbers of colonies are shown for 10^3^ (first passage colonies) or 10^4^ (second to fourth passage colonies) seeded cells. Results shown are the mean +/− SEM of 3 (MLL-CBP and MIE) or 5 (MLL-ENL) independent experiments.

To determine whether *Flt3* expression is important for the acquisition of transformed properties by primary hematopoietic cells transduced with *MLL* fusion genes, we used a myeloid progenitor replating assay [23] and compared the number of colonies generated from *Flt3* ^−/−^ or *Flt3* ^+/+^ BM cells infected by *MLL-ENL* or *MLL-CBP*. *Flt3* null mice have reduced BM pro-B cells and hematopoietic stem cells but they are healthy and display normal populations of mature hematopoietic cells [22]. In three independent experiments we found that *Flt3* genotype did not affect the ability of *MLL-ENL* or *MLL-CBP* to sustain the proliferation of colony forming cells (**Figure 1**). Moreover, *MLL-ENL* or *MLL-CBP* colonies derived from *Flt3* ^−/−^ BM displayed the same morphology and size as their *Flt3* ^+/+^ counterpart (data not shown). Cells pooled from *Flt3* ^−/−^ or *Flt3* ^+/+^ *MLL-ENL* tertiary colonies could be transferred into liquid cultures and grown for more than 6 months with cytokines, indicating that *Flt3* was not required for cell immortalization.

### *Flt3* is Dispensable to the Induction of Leukemias by *MLL* Fusion Genes

**Figure 2:**
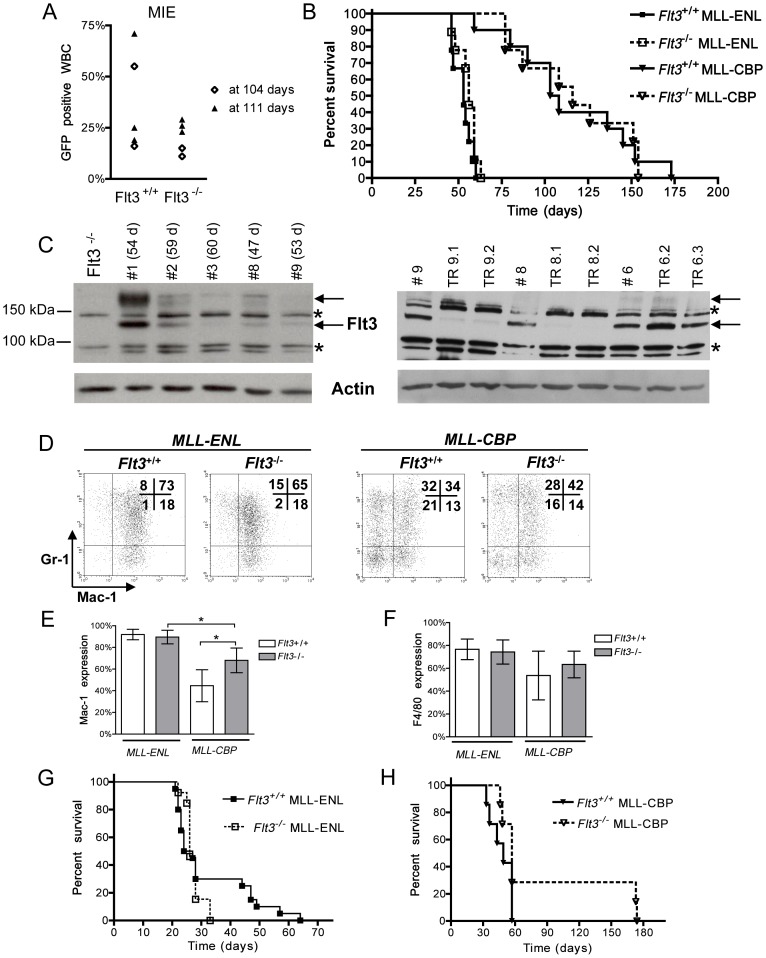
The onset and characteristics of *MLL-ENL* and *MLL-CBP* leukemias are not influenced by *Flt3* expression. **A**: Engraftment of empty vector MIE-transplanted mice measured as a percentage of GFP-expressing peripheral blood white blood cells (WBC) detected by flow cytometry. Results of 5 separate mice, transplanted with *Flt3* ^+/+^ or *Flt3* ^−/−^ MIE-transduced BM, analyzed at either 104 (diamonds) or 111 (triangles) days post-transplantation. The percentage of GFP expression in the MIE-transduced *Flt3* ^+/+^ or *Flt3* ^−/−^ BM at the time of transplantation was between 65% and 85%. **B**: Survival curves of mice transplanted with *MLL-ENL* or *MLL-CBP*-infected *Flt3* ^+/+^ and *Flt3* ^−/−^ BM (9 to 10 mice per cohort). Control mice injected with empty vector MIE-infected *Flt3* ^+/+^ or *Flt3* ^−/−^ BM remained healthy for more than 300 days. **C**: Flt3 protein levels detected by Western blot analyses of BM from primary (left panel) as well as secondary (right panel) *Flt3* ^+/+^ *MLL-ENL* leukemic mice. The survival, in days, of the primary leukemic mice is shown in parentheses (left panel). BM from a primary *Flt3* ^−/−^ *MLL-ENL* leukemic mouse is shown as a negative control in the first lane; asterisks show non-specific bands and arrows point to Flt3 bands (the upper band is glycosylated Flt3). BM protein lysates of secondary transplant mice (labeled TR) and lysates from their corresponding primary donor are loaded in adjacent lanes (right panel). Actin serves as a loading control (lower panels). **D**: Expression of Mac-1 and Gr-1 in BM blasts harvested from diseased mice. Cells were gated for GFP expression. One representative MLL-ENL and MLL-CBP mouse of each genotype are shown out of 7–8 different mice analyzed for each group. **E**: Expression of Mac-1 in BM blasts (gated for GFP expression) from leukemic mice determined by flow cytometry. Shown are average +/− SD (n = 7 to 8). *p<0.05. **F**: Expression of F4/80 in BM blasts (gated for GFP expression) from leukemic mice determined by flow cytometry. Shown are mean +/− SD (n = 7 to 8). **G**: Survival curves of secondary transplant *Flt3* ^+/+^ and *Flt3* ^−/−^ *MLL-ENL* leukemias. Graph shows survival of 13 secondary recipient mice of 5 different primary *Flt3* ^−/−^ *MLL-ENL* (each transplanted into 2 to 3 hosts) and 20 secondary recipient mice of 7 different primary *Flt3* ^+/+^ *MLL-ENL* (each transplanted into 2 to 4 hosts). **H**: Survival curves of secondary transplant *Flt3* ^+/+^ and *Flt3* ^−/−^ *MLL-CBP* leukemias. Graph shows survival of 7 secondary recipient mice of 3 different primary *Flt3* ^−/−^ or *Flt3* ^+/+^ *MLL-CBP* (each transplanted into 2 to 3 hosts).

To determine whether Flt3 is required for the onset of leukemias induced by *MLL* fusion genes, BM progenitors from *Flt3* ^−/−^ or *Flt3* ^+/+^ mice were transduced by *MLL-ENL* or by an empty MIE (MSCV-IRES-eGFP) retroviral vector and injected into lethally irradiated *Flt3* ^+/+^ recipient mice. Mice transplanted with *Flt3* ^−/−^ or *Flt3* ^+/+^ MIE-transduced BM cells remained healthy and displayed similar proportions of GFP-expressing peripheral blood leukocytes, indicating that *Flt3* deficiency did not impair BM engraftment or hematopoietic repopulating potential (**Figure 2A**). Mice transplanted with *MLL-ENL*-transduced *Flt3* ^−/−^ or *Flt3* ^+/+^ progenitors all died or became moribund and had to be sacrificed within 60 days. The survival curves of both cohorts (n = 9) were very similar (**Figure 2B**) indicating that *Flt3* did not affect leukemia latency. Moreover, considerable heterogeneity in Flt3 protein levels was seen in BM blasts collected at death from *Flt3* ^+/+^ *MLL-ENL* mice, and there was no correlation between Flt3 levels and disease latency (**Figure 2C** and data not shown). The hematological parameters of all the mice were carefully examined and are summarized in **Table 1**. All mice exhibited features of AMLs as previously described [23], [24]. These included a marked increase in number of peripheral white blood cells (WBC), splenomegaly, thrombocytopenia, invasion of BM by leukemia cells and infiltration of peripheral organs such as liver, muscle and kidneys. The only feature that distinguished *Flt3* ^−/−^ and *Flt3* ^+/+^ *MLL-ENL* leukemia mice was a reduction in leukocytosis in the absence of Flt3 (**Table 1**). The myeloid nature of the leukemias was verified by morphology assessment of peripheral blood (PB) smears and BM cytospins (data not shown). It was confirmed by flow cytometry detection of myeloid markers (Mac-1, Gr-1 and F4/80) on PB and BM blasts (**Figure 2D, E and F** and data not shown). All these characteristics were consistent with acute monocytic or myelo-monocytic leukemia and were unaffected by *Flt3* genotype. Of note, infiltration of peripheral organs by blasts was very similar in *Flt3* ^−/−^ and *Flt3* ^+/+^ *MLL-ENL* leukemias (spleen weights in **Table 1** and data not shown), indicating that Flt3 signaling is not required for migration of leukemia blasts *in vivo*.

**Table 1:** Features of AMLs induced by *MLL-ENL* or *MLL-CBP* transduction of *Flt3* ^+/+^ or *Flt3* ^−/−^ BM cells.

	Survival (days)	WBC (x10^6^/mL)	Platelets (x10^6^/mL)	Spleen weight (mg)
*Flt3* ^+/+^ *MLL-ENL*	53±6	261* ±99	187±63	393±115
*Flt3* ^−/−^ *MLL-ENL*	56±5	71* ±52	235±211	472±129
*Flt3* ^+/+^ *MLL-CBP*	115±36	133±104	277±87	375±184
*Flt3* ^−/−^ *MLL-CBP*	116±33	89±111	212±142	376±220

Values shown are mean ± SD measured for 9 to 10 mice per cohort. WBC, white blood cells.

* The WBC counts of Flt3^+/+^ and Flt3^−/−^ *MLL-ENL* mice were significantly different (p = 0.0004, unpaired t test).

To examine if Flt3 was important for leukemia transplantability, we studied the ability of *Flt3* ^+/+^ and *Flt3* ^−/−^ *MLL-ENL* leukemias to engraft into secondary recipients. BM cells from at least 5 independent primary leukemic mice of each cohort were transplanted into immunodeficient *Rag2* ^−/−^ recipient mice (2 to 4 recipients per donor). All the leukemias were transplantable and induced similar disease regardless of their *Flt3* status (**Figure 2G**, and data not shown). This suggests that *Flt3* is not essential for the self-renewal of the leukemia stem cells. Furthermore, following transplantation of two of the primary *Flt3* ^+/+^ *MLL-ENL* leukemias, propagation of leukemia cells in the recipient mice was accompanied by a reduction in Flt3 protein levels (**Figure 2C**, right panel). This provides additional evidence that leukemic blasts are not dependent on Flt3 signaling for tumoral expansion and that in addition to being dispensable for *MLL-ENL* leukemia initiation, Flt3 appears to be dispensable for leukemia maintenance.

Overall these findings demonstrate that the pathogenesis of *MLL-ENL* acute myeloid leukemias does not require Flt3 signaling. To determine whether this could be extended to another *MLL* fusion gene we repeated the transplantation experiments with BM transduced by *MLL-CBP*. We chose to study *MLL-CBP* because in contrast to other *MLL* rearrangements, it is generated by the human t(11;16)(q23;p13) which is almost exclusively associated with therapy-related myeloproliferative diseases. Accordingly, a conditional *Mll-Cbp* knockin model showed that *Mll-Cbp* could induce myeloid hyperplasia but its ability to initiate leukemogenesis required cooperating mutations [25]. This contrasts with *Mll-Enl* that rapidly induced myeloid leukemias in a chromosomal translocation model [24]. Moreover, we previously found using the retroviral transduction/transplantation model that *MLL-CBP* required a longer latency to induce AML leukemias compared to *MLL-ENL* [19]. We reasoned that under these circumstances, a lack of *Flt3* signaling might be more consequential and delay the onset or alter the phenotype of *MLL-CBP* leukemias.

In brief, we found that the latency, phenotype, and transplantability of *MLL-CBP* leukemias were essentially unaffected by the absence of *Flt3* (**Figure 2**, **Table 1** and data not shown). The only characteristic correlating with *Flt3* deficiency was an increase in monocytic markers; *Flt3* ^−/−^ *MLL-CBP* BM displayed a higher percentage of Mac-1 and F4/80 expressing blasts compared to their *Flt3* ^+/+^ counterparts (n = 7–8 mice per genotype). However, expression of these monocytic markers was highly variable and a significant difference was only seen for Mac-1 expression.

Of note, *MLL-CBP* and *MLL-ENL* leukemias are distinct clinical entities. The median survival of *MLL-CBP* mice (106 or 116 days) is two-fold longer than that of *MLL-ENL* mice (53 or 56 days). *MLL-CBP* and *MLL-ENL* leukemias have distinguishable immunophenotypes with a higher percentage of *MLL-ENL* blasts expressing monocytic (Mac-1 and F4/80) and granulocytic (Gr-1) markers compared to *MLL-CBP* blasts (data not shown and **Figure 2D–F**). Finally, in contrast to *MLL-ENL* leukemia blasts that proliferate robustly *ex vivo* in the presence of growth factors, blasts from *MLL-CBP* leukemias generally display very limited expansion in culture.

### Murine *MLL-ENL* Myeloid Leukemia Cells are Sensitive to FLT3 Inhibition *in vitro*

**Figure 3:**
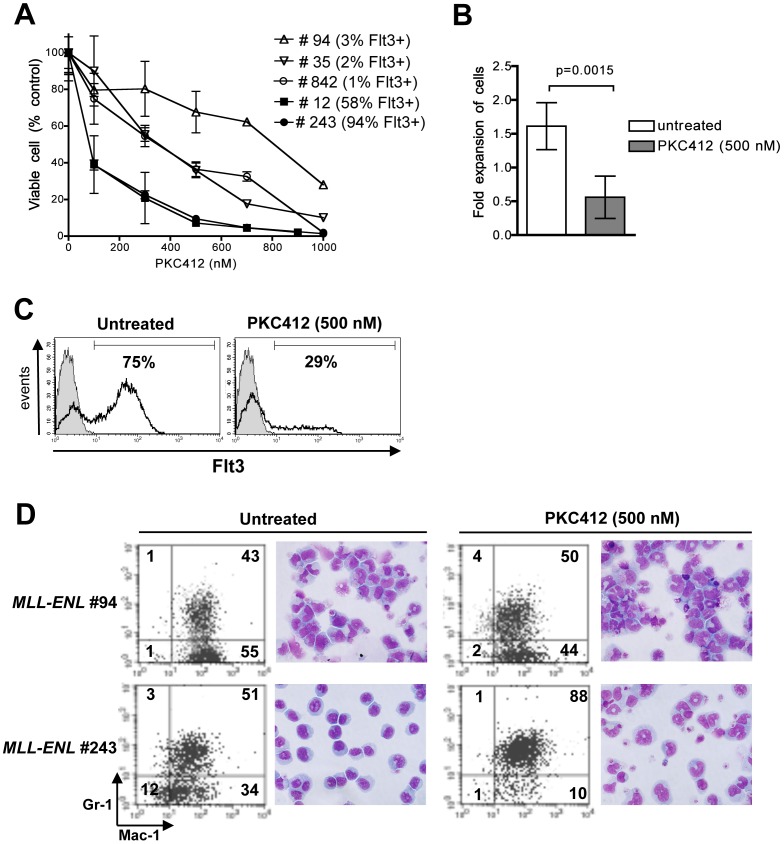
The sensitivity of *MLL-ENL* leukemia cells to PKC412 correlates with their level of Flt3 expression. **A**: Cytotoxic dose response of BM blasts from five *Flt3* ^+/+^ *MLL-ENL* mice. Viable cells were counted after 3 days of culture in the presence of increasing concentrations of PKC412, and shown as a percentage of untreated control cells. The percentage of Flt3 expressing-blasts measured by flow cytometry at the start of the assay is indicated in parantheses. Data points represent means of duplicate or triplicate wells, +/− SD**. B**: Fold expansion of viable cells during 3 days of culture (number of live cells at the end of culture divided by number of cells initially seeded) either in absence or presence of 500 nM PKC412. Mean +/− SD of results from 7 *Flt3* ^+/+^ *MLL-ENL* leukemic mice. **C**: Flt3 expression determined by flow cytometry in BM sample #243, containing initially 94% of Flt3 positive cells, measured after 3 days of culture in the absence (left) or presence (right) of PKC412 (500 nM). The gray-filled curves show non-specific fluorescence. Data is representative of 3 separate experiments. **D**: Flow cytometry analysis of Mac-1 and Gr-1 expression and morphology after 3 days of culture of samples #94 and #243 with or without PKC412 (500 nM). The percentages of cells in the quadrants are indicated. Wright Giemsa stain of cytospin preparations are shown. Similar induction of maturation was seen in blasts from 7 different *Flt3^+^* ^/+^ *MLL-ENL* leukemic mice.

To study the sensitivity of primary *MLL-ENL* blasts to the small-molecule FLT3 inhibitor PKC412, we determined the number of viable cells following incubation for 3 days with varying drug concentrations. The sensitivity of cells from distinct *MLL-ENL* leukemic mice was variable but overall PKC412 reduced viability in a dose-dependent manner with IC_50_s for most samples ranging from 100 to 700 nM (**Figure 3A**). In agreement with the heterogeneous levels of Flt3 protein seen by immunobloting (**Figure 2**), the percentage of Flt3-expressing cells measured by flow cytometry was variable among the leukemia samples. Flt3 levels did not influence the innate ability of the cells to survive/proliferate *ex vivo* as all samples displayed a similar moderate degree of expansion (approximately 1.5-fold increase in the number of viable cells during the 3-day culture period, **Figure 3B**). Incubation with PKC412 (500 nM) induced a significant reduction in cell survival with approximately half of the viable cells recovered (**Figure 3B**). Interestingly, we observed that samples containing a high percentage of Flt3 expressing cells were more sensitive to PKC412 (compare samples #243 and #12, with respectively 94% and 58% of cells expressing Flt3, to samples #94, #35 and #12 with less than 3% of cells expressing Flt3; **Figure 3A** **)**. Furthermore, in samples initially containing a high percentage of Flt3-expressing blasts, exposure to PKC412 caused an important reduction of the Flt3-positive subset (**Figure 3C**) suggesting a differential cytotoxicity of the drug towards Flt3-expressing cells. PKC412 exposure induced myeloid maturation in most samples, which could be observed by flow cytometry and morphological analyses (**Figure 3D**). Overall, these findings suggest that Flt3 targeting by PKC412 is detrimental to cell survival *ex vivo*.

### *Flt3* ^−/−^ *MLL-ENL* Leukemic Cells are Sensitive to FLT3-inhibitors in vitro

**Figure 4:**
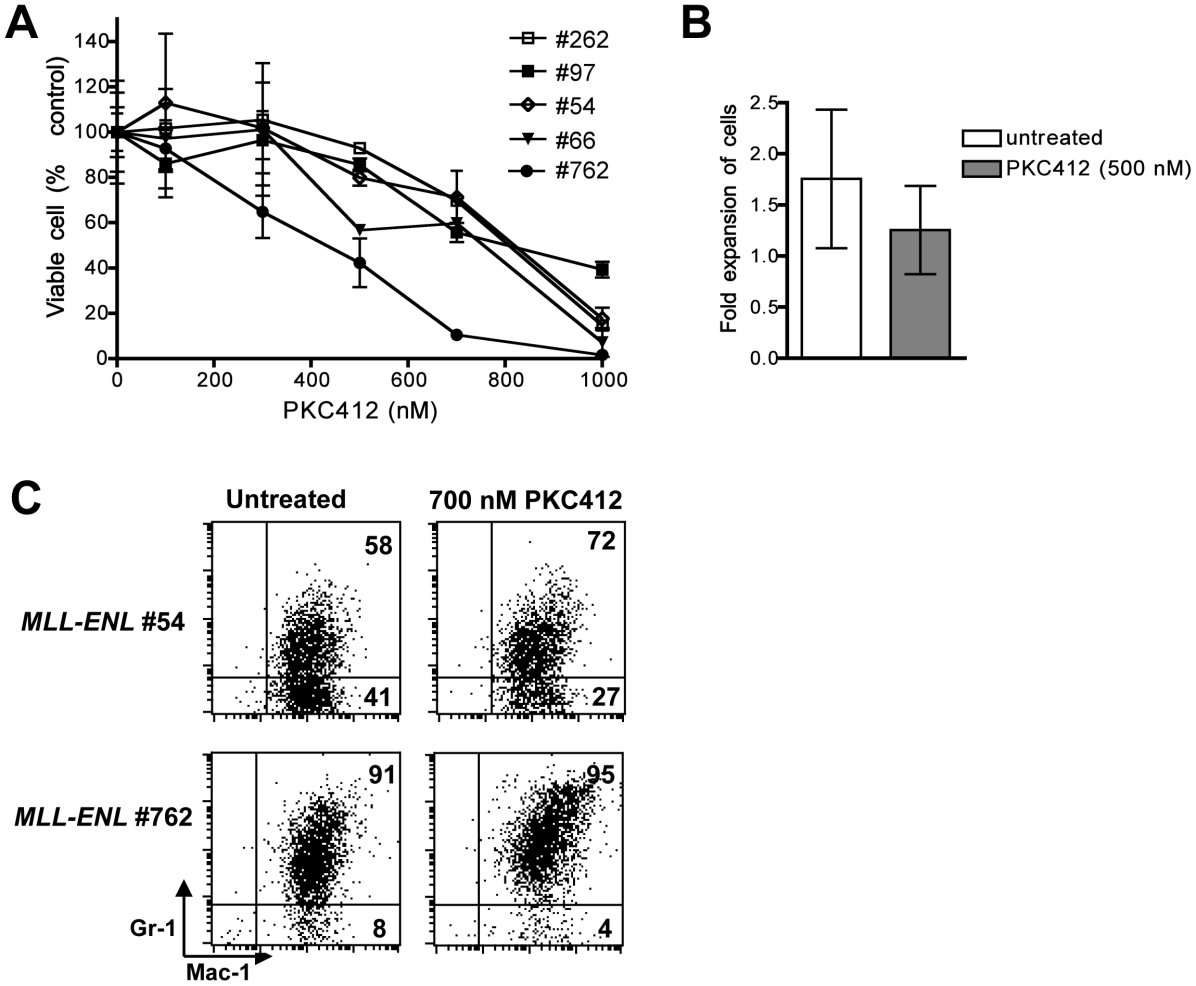
*Flt3* ^−/−^ *MLL-ENL* leukemias are sensitive to PKC412. **A** ****: Cytotoxic dose response of BM blasts from five *Flt3* ^−/−^ *MLL-ENL* leukemic mice. Viable cells were counted after 3 days of culture in presence of increasing concentrations of PKC412 and shown as percentage of untreated control cells. Data points represent means of duplicate or triplicate wells, +/− SD. **B**: Fold expansion of viable cells during 3 days of culture (number of live cells at the end of culture divided by number of cells initially seeded) either in absence or presence of 500 nM PKC412. Mean +/− SD of results from 6 *Flt3* ^−/−^ *MLL-ENL* leukemic mice. **C**: Flow cytometry analysis of Mac-1 and Gr-1 expression after 3 days of culture of samples #54 and #762 either without or with PKC412 (700 nM). The percentages of cells in the quadrants are indicated. Similar induction of maturation was seen in blasts from 5 different *Flt3^−/−^ MLL-ENL* leukemic mice.

To explore whether the toxicity of PKC412 towards *MLL-ENL* leukemia cells was solely mediated by Flt3 inhibition, we studied the sensitivity of *Flt3* ^−/−^ *MLL-ENL* leukemias. Flt3 deficient cells displayed a dose-response to PKC412 with IC_50_s ranging from 400 to 800 nM (**Figure 4A**); this range was similar to the IC_50_ measured for *MLL-ENL Flt3* ^+/+^ cells expressing low levels of Flt3 (samples #94, #35, and #842 in **Figure 3A**). Again we found that exposure of *Flt3* ^−/−^ blasts to PKC412 induced myeloid maturation visible by a shift in Mac-1 and Gr-1 expression (**Figure 4C**) and changes in morphology (data not shown).

## Discussion

Our objective was to determine the role of Flt3 in the pathogenesis of *MLL*-rearranged leukemias and to use a murine leukemia model to study the sensitivity of blasts to FLT3 inhibition *ex vivo*. Using genetically modified mice lacking *Flt3* as a source of hematopoietic precursors, we found that Flt3 signaling was dispensable for *in vitro* and *in vivo* oncogenic transformation by *MLL-ENL* or *MLL-CBP*. With either fusion genes, leukemia onset and progression, infiltration of peripheral organs or leukemia transplantability were unchanged in the absence of Flt3. The presence of Flt3 did have a notable effect on leukocytosis with a considerable reduction in circulating blasts in *Flt3* ^−/−^ MLL-ENL leukemias compared to their *Flt3* ^+/+^ counterparts. This did not seem to be caused by a lesser proliferation rate of *Flt3* ^−/−^ *MLL-ENL* blasts as it was not accompanied by reduced leukemia infiltration of organs or longer disease latency, and the proliferation potential of the *Flt3* ^−/−^ and *Flt3* ^+/+^ *MLL-ENL* blasts was comparable upon *ex vivo* culture (**Figures 3B** and **4B**). The lower number of circulating blasts may instead be connected with Flt3’s known role in controlling migration of hematopoietic precursors from the BM to the peripheral blood [26]. This property could also explain the effectiveness of FLT3 inhibitors in lowering peripheral blood blasts seen in clinical trials [13].

Interestingly a spontaneous reduction of Flt3 expression upon transplantation of leukemia cells to secondary recipient mice was seen with two independent primary *MLL-ENL* leukemias (**Figure 2C**). This indicates that sustained Flt3 expression does not confer a selective advantage to expanding leukemic clones *in vivo*. It also indicates that in addition to being dispensable for the initiation of myeloid leukemias, Flt3 is dispensable for leukemia maintenance. This contrasts with a report showing that shRNA-mediated knock down of Flt3 significantly, albeit moderately, delayed the onset of *MLL-AF9* AMLs in transplanted mice [11]. The reasons for this discrepancy are unclear but we cannot rule out differences in Flt3 requirement between *MLL-AF9* and *MLL-ENL* or *MLL-CBP* leukemias.

The sensitivity of primary human *MLL*-rearranged ALL samples to FLT3 inhibitors was shown to be proportional to the level of FLT3 expression [14], [15]. In line with this, our data with murine *MLL-ENL* AMLs also show that PKC412’s cytotoxicity is more pronounced toward samples expressing higher levels of Flt3. This argues that PKC412’s cytotoxicity is mediated by Flt3 inhibition and that the Flt3 pathway contributes to cell survival in culture. However, this is at odds with previously published *in vivo* studies of FLT3 inhibitors performed with either xenotransplants of human cell lines [7] or transplantation of retrovirally transduced mouse BM [18]. In these investigations the therapeutic efficacy of PKC412 was restricted to *MLL*-rearranged leukemia cells carrying either *FLT3* amplifications or FLT3 activating mutations; whereas no therapeutic benefit could be observed in cells in which *FLT3* was unaltered [7], [18]. This suggests that in contrast to FLT3 activating mutations or amplifications, FLT3 signaling resulting from overexpression of wild type FLT3 may not cause a proliferative advantage to *MLL*-rearranged leukemias *in vivo*; this is consistent with our findings that *Flt3* deficiency does not delay *MLL*-rearranged leukemias. These findings highlight a disparity between the absence of Flt3 dependency of *MLL*-rearranged leukemias *in vivo* and their sensitivity to FLT3 inhibition *ex vivo*. Two explanations can be proposed to address this paradox. First, primary leukemia cells adopt very different biological properties when they are cultured *ex vivo*, notably their growth slows down considerably. Hence Flt3 signaling might be important for cell survival *ex vivo* while being dispensable to the proliferation of leukemic cells *in vivo*, conceivably because of abundant stimuli in the bone marrow environment. Second, PKC412 while being more active towards cells expressing high levels of Flt3, also exerts its cell toxicity through the combined inhibition of other molecular targets. Like most small molecule kinase inhibitors that target the ATP binding site, PKC412 inhibits multiple kinases other than FLT3 (including c-KIT, PDGFR and VEGFR-2). The dose response cytotoxicity and induction of differentiation seen in *Flt3*-deficient cells is evidence of PKC412’s off-target activity. This ability to inhibit multiple targets may play a considerable role in the anti-leukemic activity of FLT3 inhibitors. This is supported by studies of primary human *MLL*-rearranged leukemia cells in which PKC412 doses required for repression of cell growth were considerably higher than those sufficient to fully inhibit FLT3 phosphorylation [15]. Another report comparing several FLT3 inhibitors on primary AML samples also concluded that less selective compounds were most effective at inducing cytotoxicity [27]. Conversely, highly specific strategies like siRNA or inhibitory monoclonal antibodies directed against particular kinases might be less efficient.

The discrepancy between our *in vitro* and our *in vivo* findings highlights the need to incorporate several experimental models in preclinical evaluation of novel drugs. *In vitro* assays are inherently more artefactual. For instance we observed that increasing fetal bovine serum content or adding cytokines to the culture media greatly influenced the cells’ sensitivity to PKC412 (data not shown). The murine *in vivo* assay also has caveats in recapitulating the human *MLL*-rearranged leukemias. Although we showed that Flt3 was dispensable for the generation of leukemias induced by two distinct *MLL* fusion genes, both diseases were myeloid malignancies and we cannot rule out that *FLT3* may be important in the pathogenesis of *MLL*-rearranged lymphoid malignancies. However, identification of a common gene expression signature of *MLL*-rearranged leukemias regardless of lineage [4], [28] suggests that mechanisms underlying *MLL* fusion gene-induced ALLs and AMLs are, to some extent, conserved. The ultimate evaluation of the therapeutic benefit of FLT3 inhibitors against *MLL*-rearranged leukemias awaits results of clinical trials in patients. Their analysis should also determine whether FLT3 inhibition per se or off-target activity mediates the efficiency of these compounds.
